# IgE-tailpiece associates with α-1-antitrypsin (A1AT) to protect IgE from proteolysis without compromising its ability to interact with FcεRI

**DOI:** 10.1038/srep20509

**Published:** 2016-02-04

**Authors:** Phyllis M. Quinn, David W. Dunne, Shona C. Moore, Richard J. Pleass

**Affiliations:** 1 Institute of Genetics, School of Biology, University of Nottingham, Nottingham NG7 2UH, UK; 2 Department of Pathology, University of Cambridge, Tennis Court Road, Cambridge CB2 1QP, UK; 3 Department of Parasitology, Liverpool School of Tropical Medicine, Pembroke Place, Liverpool, L3 5QA, UK; 4 Warwick Systems Biology Centre, Senate House, University of Warwick, Coventry CV47 7AL, UK

## Abstract

Several splice variants of IgE exist in human plasma, including a variant called IgE-tailpiece (IgE-tp) that differs from classical IgE by the replacement of two carboxy-terminal amino acids with eight novel residues that include an ultimate cysteine. To date, the role of the secreted IgE-tp isoform in human immunity is unknown. We show that levels of IgE-tp are raised in helminth-infected donors, and that both the classical form of IgE (IgE-c) and IgE-tp interact with polymers of the serine protease inhibitor alpha-1-antitrypsin (A1AT). The association of IgE-tp with A1AT polymers in plasma protects the antibody from serine protease-mediated degradation, without affecting the functional interaction of IgE-tp with important receptors, including FcεR1. That polymers of A1AT protect IgE from degradation by helminth proteases may explain why these common and normally non-disease causing polymorphic variants of A1AT have been retained by natural selection. The observation that IgE can be complexed with polymeric forms of A1AT may therefore have important consequences for our understanding of the pathophysiology of pulmonary diseases that arise either as a consequence of A1AT-deficiency or through IgE-mediated type 1 hypersensitivity responses.

Immunoglobulin E (IgE) functions by binding to IgE-receptors (FcεRI and FcεRII) found on the surfaces of immune cells, including basophils and mast cells, which when activated in the lung induce the release of toxic mediators responsible for the symptoms of asthma^1^,^2^,^3^. IgE can also bind FcεRI expressed by monocytes and dendritic cells, where it is believed to promote the development and activation of Th2 cells thereby contributing to allergic inflammatory disease^4^. However, recent studies have shown that FcεRI on DCs and monocytes contributes to serum IgE clearance and is involved in restraining inflammation at mucosal sites^5^,^6^,^7^,^8^.

Only one species of secreted IgE was thought to exist until the discovery of several isoforms generated by alternative splicing of the human Cε gene^9^,^10^,^11^,^12^,^13^,^14^,^15^. One of these, termed IgE-tailpiece (IgE-tp), differs from classical IgE (IgE-c) in possessing an eight amino acid carboxy-terminal tailpiece that terminates in a cysteine residue, whose function remains enigmatic. Messenger RNA for IgE-tp has been observed in all IgE positive cell sources examined, including cell lines, fresh peripheral blood leukocytes stimulated with IL4/anti-CD40, as well as spontaneous IgE producing B cells from hyper-IgE syndrome deficiency patients^10^,^11^,^12^. Limited studies have shown that tailpiece specific anti-sera can detect native protein from human IgE secreting cell lines^10^,^11^, sera from IgE myeloma patients, and plasma from both normal and atopic individuals^9^,^11^,^12^. That the IgE population in humans is not homogenous is confirmed from functional studies of IgE involving sera from atopic patients showing that only half of these individuals possessed IgE that can passively sensitize basophils from normal individuals and trigger histamine release^16^. Furthermore, the observation of several bands both in Northern and Western blotting, supports the notion of heterogeneity in the IgE family^17^.

Levels of IgE-tp are unchanged in atopy and may not be a major determinant of allergic inflammation^18^. Although recombinant IgE-tp has been shown to bind with equal affinities to both the FcεRI and FcεRII and possesses similar biological properties when compared with the classical form of secreted IgE (IgE-c)^9^,^14^, there is some evidence that *in vivo* this variant may interact with plasma proteins that may bestow unique immunological functions on this IgE variant^9^.

Parasitic helminth infections, including those responsible for the disease schistosomiasis, are also associated with high titers of specific and non-specific IgE antibody and many reports have shown an important role for human IgE in parasite killing^19^,^20^,^21^, although a role for IgE-tp has yet to be investigated in worm infections. Most clinically important human helminth parasites interact with IgE in respiratory tissues as a consequence of their scripted migratory life cycles^22^,^23^. The migration through the lungs results in lasting changes to the immunologic, physiologic and structural architecture of the lungs that result in focal damage to the epithelium giving rise to emphesema-like pathology and symptoms^22^,^24^.

Human IgE and IgE-tp are efficiently degraded *ex-vivo* by helminth and human serine proteases that cleave IgE in the Fc, resulting in IgE molecules that are unable to interact with Fcε-receptors^25^,^26^. However, evidence of IgE cleavage occurring *in vivo* could not be found, and we therefore speculated that human IgE associates with another plasma protein to protect it from serine-protease mediated degradation. Here we show that IgE and IgE-tp interact with plasma alpha1-antitrypsin (A1AT), encoded by the serpin peptidase inhibitor, clade A gene (SERPINA1). The interaction with A1AT protects IgE from cleavage by serine proteases and enhances interactions with FcεR1 expressed by fibroblasts. Although the mechanism(s) for the increased interactions with FcεR1 observed here have not been elucidated, they nonetheless point to a potentially important role for IgE in modifying A1AT mediated clearance of pathogenic A1AT polymers and serine proteases.

## Results

### Levels of IgE and IgE-tp are raised in helminth-infected donors and exist as high molecular weight forms in plasma

**Figure 1:**
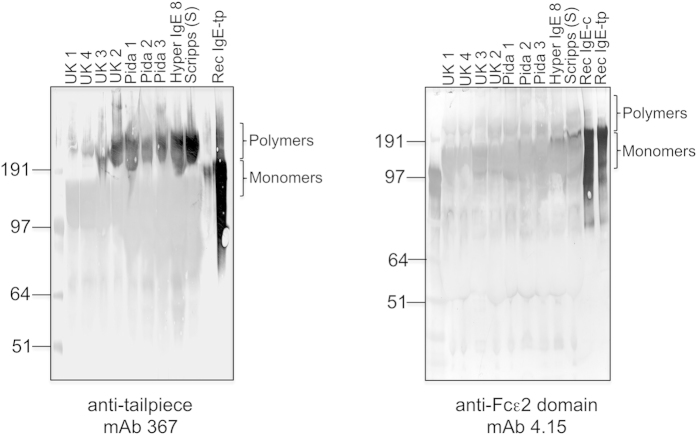
IgE from human plasma runs as polymers as well as monomers. Western blot analysis of plasma samples from UK healthy donors, Ugandans from Pida where schistosomiasis is endemic, a Scripps hyper IgE plasma sample and internal standard, recombinant IgE-c or IgE-tp described previously^9^,^15^. Five microliters of plasma were run under non-reducing conditions and probed with either anti-tailpiece (367) or anti-Fcε2 domain (4.15) specific monoclonal antibodies.

We previously demonstrated that human IgE and IgE-tp are susceptible to cleavage *ex-vivo* by proteases released from *S. mansoni*^25^,^26^. To investigate if cleavage also occurred *in situ*, we screened numerous plasmas derived from helminth-infected donors for the presence of degraded IgE and IgE-tp (Fig. 1). No low-molecular weight fragments (26 and 13 kDa) that are indicative of IgE-tp or IgE-c cleavage also occurring *in vivo* were observed. On the contrary, the tailpiece-specific mAb 367 and an anti-ε2 mAb (4.15) detected IgE at varying high molecular weights (HMW) of approximately 250–400 kDa, as determined by their relative electrophoretic mobility compared with the monomeric recombinant IgE-c and IgE-tp controls which run at the expected molecular weight for IgE of 190 kDa (Fig. 1). The observations of HMW IgE-types in plasma are in agreement with a previous study^9^. As expected for an alternatively spliced product, levels of IgE-tp were generally also raised in individuals with high pre-existing IgE titers, including individuals from a Ugandan population where the prevalence of helminth infection was 72%^27^ (Fig. S1).

### IgE binds alpha-1-antitrypsin (A1AT)

The HMW forms of IgE-tp may represent complexes with another plasma protein that protects IgE from cleavage by schistosome proteases that are commonly chymotrypsin-like^26^. Given the propensity of α1-antitrypsin (A1AT) to neutralize serine proteases together with the observation that A1AT is known to exist in covalent complexes with κ-light chains^28^ and other tailpiece-containing IgA antibodies^29^, we hypothesized that A1AT may be a candidate for binding to IgE-tp. We initially investigated binding of IgE to A1AT by ELISA (Fig. S2). We observed that both recombinant IgE-c and IgE-tp (both containing identical κ-light chains) could bind to A1AT coated to ELISA plates (Fig. S2), a finding confirmed by surface plasmon resonance analysis (SPRA) with recombinant IgE-tp and a commercially available preparation of monomeric A1AT (Fig. S3).

**Figure 2:**
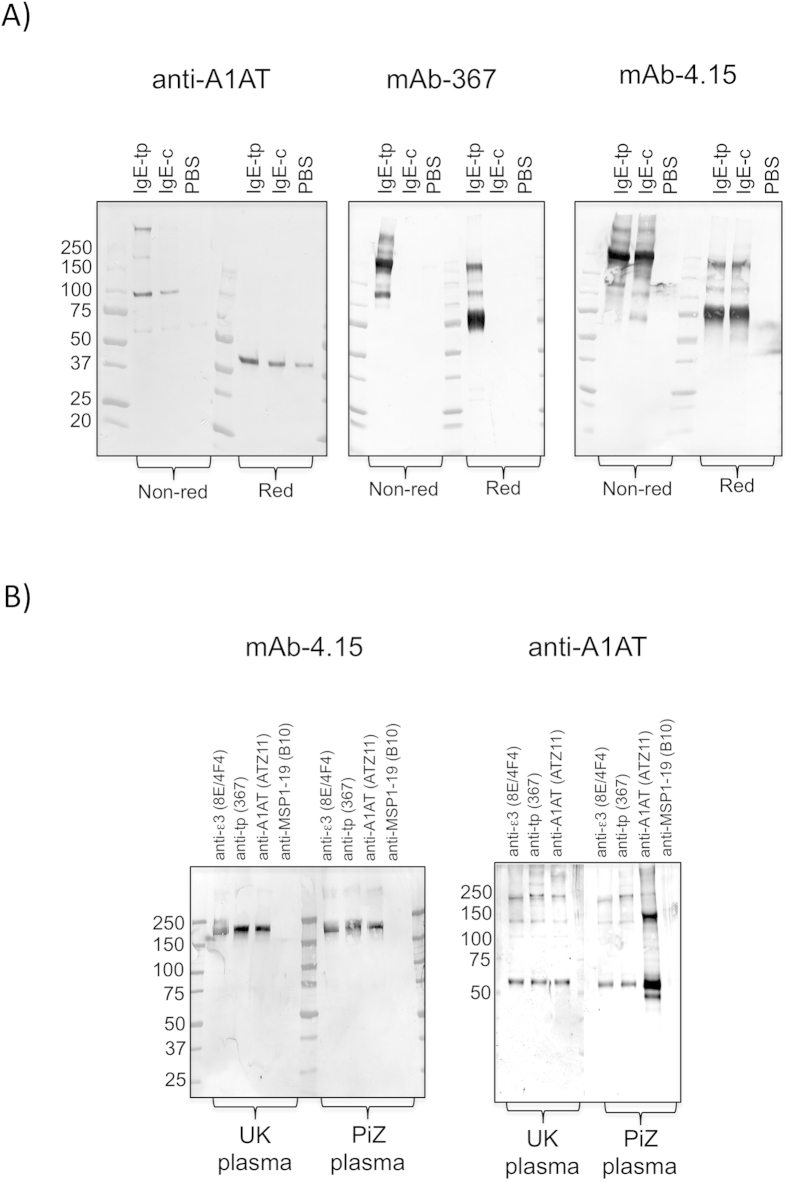
Recombinant IgE-c and IgE-tp associate non-covalently with A1AT in plasma. (**A**) Immunoprecipitation of A1AT from human plasma by IgE-c or IgE-tp run under non-reducing (Non-red) or reducing (Red) conditions. (**B**) Reciprocal precipitations of IgE from either UK or PiZ plasma with monoclonal antibody ATZ11 specific for polymers of A1AT. Complexes were probed with either anti-tailpiece (367), anti-Fcε2 (4.15), or anti-A1AT antibodies.

To verify our ELISA and SPRA observations we precipitated A1AT directly from human plasma using recombinant human IgE-c or IgE-tp antibodies as bait (Fig. 2A). We could show that both IgE-c and IgE-tp were able to pull-down A1AT from normal human plasma when affinity purified using dansyl-BSA-conjugated beads, the hapten recognized by both the recombinant IgE monoclonal precipitating antibodies (Fig. 2A). The IgE-tp pulled down numerous A1AT reactive bands of approximately 100 kDa, 200 kDa, and >250 kDa, possibly reflecting dimers, tetramers and higher order multimers of A1AT which is known to form sequential circularizing polymers on heating^30^,^31^,^32^. The multimeric forms of A1AT collapsed to a single-band of ~53 kDa on reduction, indicating that A1AT polymerization was dependent on covalent interactions within A1AT but not with IgE, as seen with IgA-A1AT complexes^33^.

Western blotting of the precipitated complexes with the anti-IgE-tp (monoclonal 367) or an anti-IgE-Fc confirmed that: i) IgE was also present in the complex, and ii) that the interaction with A1AT caused no intrinsic degradation to either IgE or IgE-tp. The formation of the tetramer and >250 kDa species of A1AT seen with IgE-tp was reliant on the 8 amino acid C-terminal tailpiece of this IgE isoform, since prior incubation with the tailpiece-specific monoclonal antibody 367 blocked their formation (Fig. S4), whilst not affecting the binding of the 100 kDa A1AT form. This shows that A1AT binding to IgE-tp occurs at two distinct locations on the molecule, one location for dimer forms of A1AT shared with IgE-c and another for larger polymers of A1AT that is dependent on the eight amino acid C-terminal tailpiece which is absent from IgE-c.

To be more certain of the interaction between plasma IgE and A1AT, we next used monoclonal ATZ11 raised against the most common Z form variant of A1AT to co-precipitate natural IgE from plasma (Fig. 2B). ATZ11 recognizes a conformation dependent neo-epitope created when A1AT multimerizes or complexes with its target proteases^34^. Using ATZ11 we could co-precipitate IgE from normal UK or *PiZ* (protein inhibitor homozygous ZZ allele E342K) plasma that was recognized by the anti-IgE Cε2 monoclonal 4.15 or anti-A1AT antibodies (Fig. 2B).

The neo-epitope recognized by ATZ11 is also clearly available when A1AT associates with IgE and occurs irrespective of plasma coming from healthy controls or *PiZ* donors as described previously^34^. Reassuringly, ATZ11 bound more A1AT from *PiZ* plasma than from normal donors (Fig. 2B). The complementary experiment using mAbs 8E/4F4 (recognizing the Cε3 constant domain of human IgE-Fc) or mAb 367 (recognizing the tailpiece) also pulled down A1AT irrespective of donor. Taken together these experiments clearly show that IgE interacts with A1AT in human plasma.

### Marked variability in IgE binding seen with A1AT variants associated with A1AT deficiency and disease

The *PiZ* variant, characterized by an E342K substitution is the most common and clinically relevant mutation responsible for A1AT deficiency^35^. A1AT from individuals homozygous for this point mutation is prone to polymerize through different mechanisms including RCL insertion and/or C-terminal domain swapping^30^,^31^,^32^,^36^. Such polymers aggregate in the endoplasmic reticulum of hepatocytes, with resultant plasma deficiency, predisposing these individuals to chronic obstructive pulmonary disease (COPD) as a consequence of excess lung damage by neutrophil elastase.

**Figure 3:**
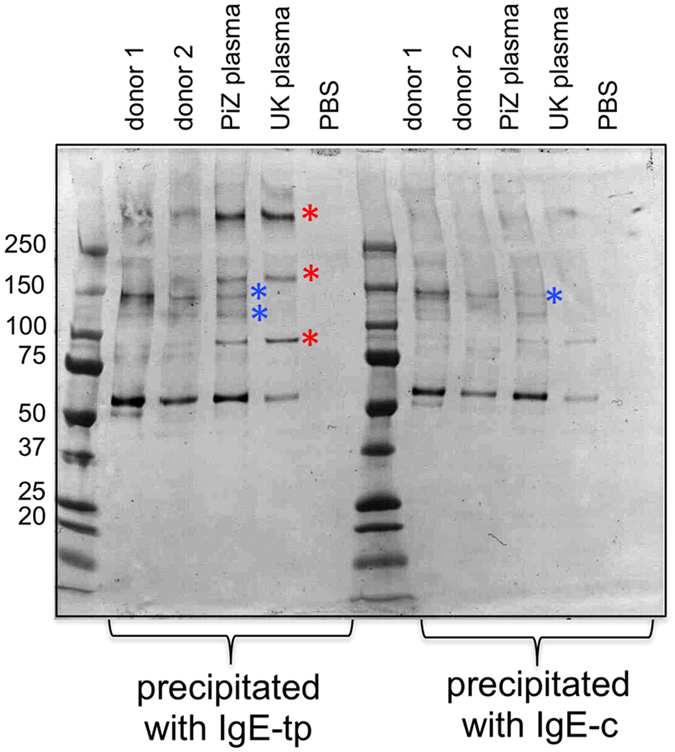
Human A1AT from patients with COPD or PiZ phenotype still bind IgE. Human A1AT from three patients with COPD (chronic obstructive pulmonary disease) form A1AT polymers that run at different molecular weights to those seen with healthy donors that can still bind IgE. A1AT was precipitated from human plasma with either IgE-c or IgE-tp and probed with anti-A1AT antibodies.

We hypothesized that polymorphic variants of A1AT, including the *PiZ* variant, may therefore result in forms of A1AT that are less able to interact with IgE. To address this question we screened plasmas from three individuals with chronic COPD, including a known *PiZ* typed variant for binding to IgE (Figs 2 and 3). Although A1AT from *PiZ* plasma was as good at associating with IgE-tp and multimerizing as A1AT from healthy donors, A1AT from two other donors were significantly impaired in their ability to bind and form A1AT multimers with IgE-tp, that are of the same molecular weight to those seen in healthy plasma (Fig. 3, indicated with red stars). Indeed, A1AT from all three individuals bound IgE although some of these polymers were not seen in pull-downs with normal plasma (Fig. 3, indicated with blue stars). For example a trimer species running just short of 150 kDa could be precipitated by IgE-c from all three patients that was not seen with healthy donors. The findings show that variants of A1AT that are found in plasma from individuals with COPD can still interact with IgE and IgE-tp but that that the nature of the oligomeric forms arising are different.

### IgE-tp partially triggers A1AT polymerization in the absence of other plasma components

To determine if IgE could trigger the polymerization of A1AT in the absence of other serum components or heating, we incubated equimolar concentrations of a commercially available A1AT preparation with recombinant IgE-tp. After an 18 h incubation at room temperature in the presence of IgE-tp, A1AT only partially formed polymers that were of an equivalent molecular weight to those derived by immune-precipitation from plasma directly (Fig. S5A). Because SDS-PAGE results in the dissociation of A1AT from IgE we subjected these incubations of IgE-tp with A1AT to analysis by size-exclusion chromatography (Fig. S5B). This revealed that both A1AT and IgE-tp run as monomers, with approximate molecular weights of 55 and 190 kDa, and that therefore the two molecules were not in complex with one another. This suggests that either IgE must be complexed with antigen for binding of A1AT, or another protein or other conditions provided by plasma but absent from these incubations with highly purified monomeric proteins, may assist in the interaction of IgE-tp with A1AT *in vivo*.

### Cleavage of the reactive centre loop (RCL) of A1AT inhibits the formation of polymers capable of interacting with IgE

**Figure 4:**
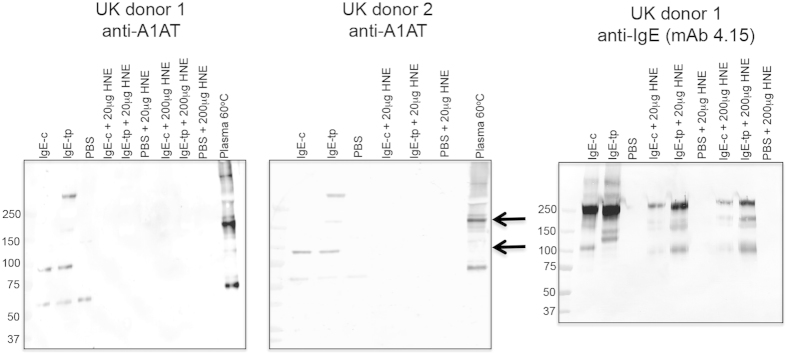
Cleavage of the reactive centre loop (RCL) of A1AT by human neutrophil elastase (HNE) results in molecular forms of A1AT that no longer bind IgE. Plasma from two different healthy UK donors were incubated with either PBS (controls) or 20 μg and 200 μg HNE prior to precipitation with IgE-c or IgE-tp as indicated. Plasma from each donor was also heated at 60 °C for 20 min to recapitulate features of A1AT polymers associated with disease and retention of A1AT in the liver. All samples were run under non-reducing conditions and probed with anti-A1AT or anti-IgE Fcε2 domain (4.15) specific monoclonal antibodies.

To further investigate the nature of the bound A1AT we wondered if A1AT was capable of binding IgE or of forming sequential polymers after it had first interacted with its target enzyme, a process resulting in cleavage and irreversible insertion of the RCL into β-sheet A of A1AT^37^,^38^. To test this hypothesis we first incubated plasma with human neutrophil elastase (HNE), the target substrate for A1AT, and an interaction resulting in irreversible cleavage of the RCL of A1AT, prior to incubation with either IgE-c or IgE-tp. Complete inhibition of the formation of polymeric forms of A1AT capable of binding IgE was observed after cleavage of the RCL of A1AT by HNE (Fig. 4). This shows that binding of A1AT to IgE is dependent on a fully functional RCL, and that only the non-protease bound conformational state of A1AT could bind IgE. The multimerized forms of A1AT generated by heating plasma, a process believed to recapitulate features of polymers associated with disease, are clearly different in their amount and type to those that form after association with IgE. For example, trimers of A1AT are more readily seen than dimers when plasma is heated directly (Fig. 4, arrowed). Overall these finding show that, i) gross conformational changes in A1AT as a consequence of inactivating its target enzyme results in A1AT molecules no longer capable of interacting with IgE, and that ii) the forms of A1AT that interact with IgE are subtly different to those induced by heating plasma directly^39^,^40^,^41^.

### Binding of A1AT by IgE-tp protects it from proteolysis

**Figure 5:**
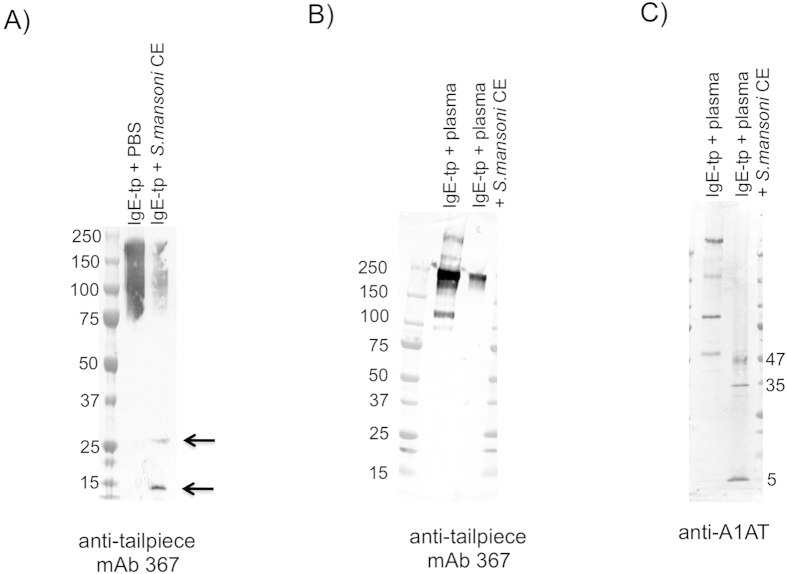
Pre-incubation of IgE-tp with plasma as a source of A1AT prevents degradation of IgE-tp by schistosome cercarial elastases (CE). (**A**) In the absence of A1AT provided in plasma, IgE-tp is cleaved by CE at the Cε3/Cε4 junction into two low molecular weight cleavage products of 26 and 13 kDa respectively that can be detected with anti-tailpiece monoclonal antibody 367. (**B**) The cleavage products detected by 367 are lost with prior incubation of IgE-tp in plasma showing that the A1AT associated with IgE prevented the breakdown of IgE by CE. Intact IgE-tp runs with an approximate molecular weight of 195 kDa (**C**) Staining with anti-A1AT revealed that the polymeric forms of A1AT associated with IgE are inactivated by CE. The resulting conformational change in the A1AT-enzyme complex is observed by the radical change in molecular weight to bands of 47, 35, and 5 kDa that are indicative of inactivated A1AT complexed with serine proteases as documented previously^42^,^63^.

Human IgE is known to be highly susceptible to cleavage and inactivation by trypsin and elastase proteases derived from parasitic helminths, the target substrates for A1AT^38^. We therefore investigated if the interaction of A1AT with IgE protects the antibody from proteolytic degradation by these types of enzyme. To investigate this possibility we used mAb 367 recognizing the C-terminal eight amino acid tailpiece as a tag to track cleavage within the IgE molecule as previously described^26^ (Fig. 5). When IgE-tp is digested by schistosome proteases, the cleaved Cε4 domain is detected as two products of ~13 and 26 kDa consistent with cleavage occurring within the Fc^26^ (Fig. 5, panel A arrowed). When IgE-tp is pre-incubated in plasma and A1AT allowed to bind, these cleavage products cease to be visible (Fig. 5, panel B). This shows that A1AT protects IgE from enzymatic degradation. Although the IgE remained intact, blotting with anti-A1AT antibodies revealed that the A1AT carried by IgE had changed radically, indicating that they had reacted with the provided proteases and thereby protecting the IgE molecule from degradation. Molecular weights of bands seen at 47, 35 and 5 kDa are consistent with the formation of protease A1AT complexes (Fig. 5, panel C) as described previously^42^. This data clearly show that IgE piggybacks A1AT to protect it from breakdown by proteases.

### Binding of A1AT by IgE does not block interactions with the high affinity Fcε-receptor I (FcεRI)

**Figure 6:**
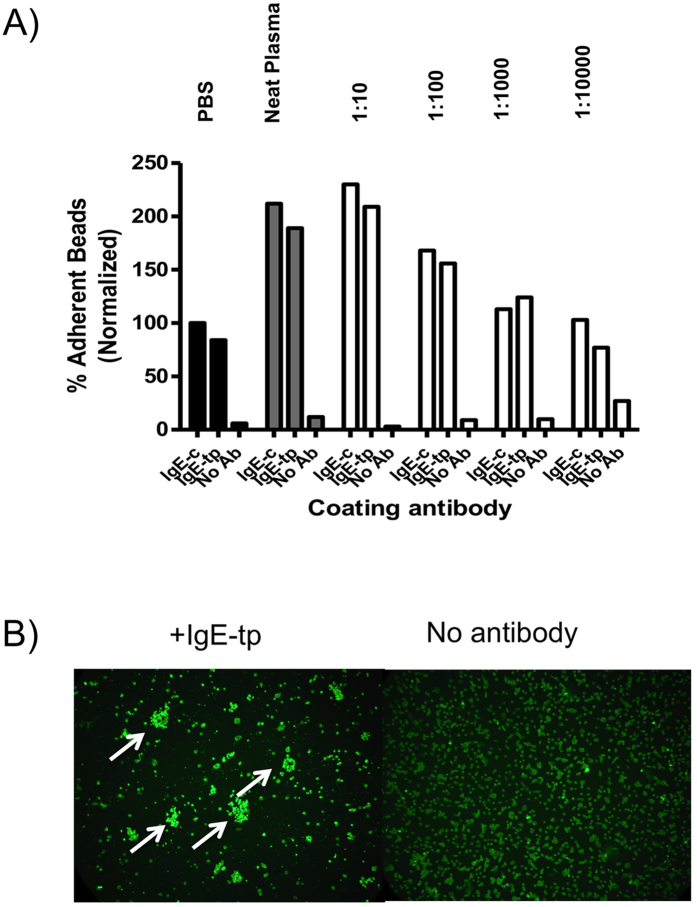
IgE interactions with the high-affinity Fc R1 are strengthened by prior incubation of IgE-opsonized beads with human plasma. (**A**) Histograms of specific binding of IgE-tp and IgE-c with the Fc RI transfected CHO cell line CHK1E1. Results are normalized by expressing adherence of IgE-opsonized beads as a percentage of adherence seen with IgE-c in the absence of plasma. (**B**) Binding can also be visualized as rosettes of CHK1E1 around IgE-tp opsonized beads detected with an anti-FcεR1-FITC antibody used to confirm expression of FcεR1 by the transfected cell line.

We next investigated if binding of A1AT to IgE may interfere with the function of IgE, perhaps by blocking interactions with IgE Fc-receptors. To test this hypothesis, dansyl-sepharose beads opsonized with IgE from plasma incubations were tested for their ability to bind CHK1E1 cells expressing the α and γ subunits of the high affinity IgE receptor, FcεRI (Fig. 6). The CHK1E1 thus provides a direct system for studying the ability of IgE-A1AT complexes to modulate the binding of IgE to FcεR1^43^. To our surprise, prior incubation of IgE with neat plasma doubled rosette formation that diminished with increasing dilutions of plasma (Fig. 6). This result shows that IgE bound to A1AT does not inhibit the interaction of IgE with FcεRI, but rather enhances it.

## Discussion

We have previously shown that IgE is rapidly cleaved by serine proteases of both parasite and host origin^26^. To look for evidence of cleavage occurring *in vivo*, we screened plasmas from hyper-IgE and helminth-infected donors for the presence of degradation products using Fcε-specific monoclonal antibodies (367 and 4.15). Although raised levels of IgE and IgE-tp were observed in both donor cohorts compared with healthy controls (Fig. S1), no evidence for the occurrence of IgE degradation to low molecular weight fragments as observed with *ex vivo* cleavage was found (Fig. 1). On the contrary, we consistently observed the presence of high molecular weight (HMW) forms of IgE-tp that were much larger (250–400 kDa) than that predicted for single IgE molecules (~190 kDa), and in agreement with an earlier study^9^.

The carboxy-terminal cysteine located at the end of the eight amino acid tailpiece of IgE-tp is positioned differently to the penultimate cysteine found on IgA and IgM tailpieces, that are utilized during polymer formation of these isotypes^44^,^45^. However, the recombinant IgE-tp expressed by transfected Sp2/0 mammalian cell lines did not run as polymers (Fig. 1A 9,15), raising the possibility that IgE-tp multimerization *in vivo* may be facilitated by another human serum protein that forms covalent interactions with IgE-tp. For example, the IgA molecule is able to form covalent complexes with A1AT that is dependent on disulphide bonding between Cys232 on A1AT and the penultimate Cys471 of the IgA Cα3 domain^33^. These IgA-A1AT complexes are found at raised levels in the sera and synovial fluid of patients with rheumatoid arthritis^29^ and ankylosing spondylitis^46^. We therefore wondered if A1AT was a target for IgE-tp that could explain our observation of HMW complexes seen in plasma.

Since recombinant monoclonal IgE-tp molecules clearly run as monomers (and are therefore not complexed to a partnering protein)^9^,^15^, and taking advantage of its affinity for dansyl-BSA, we were able to pull down A1AT in complex with IgE-tp, and perhaps more surprisingly also with the classical form of IgE that does not contain the tailpiece cysteine (Fig. 2A). Human A1AT is known to form sequential circular polymers^30^, and the forms of A1AT that bound IgE-tp ran with molecular weights of ~100, 200, and >250 kDa that possibly reflect these sequential dimers, tetramers and higher order oligomeric forms of A1AT. To our surprise the IgE control pulled down A1AT that formed 100 kDa dimers, indicating that the binding site on IgE giving rise to this dimeric form of A1AT did not involve the tailpiece. Furthermore pre-incubation of recombinant IgE-tp with the tailpiece specific monoclonal 367 blocked the formation of HMW forms of A1AT while leaving the 100 kDa dimer species intact (Fig. S4). It is thought that A1AT dimers are an important intermediate in propagating polymer formation^47^. This shows that A1AT binds at more than a single site on the IgE-tp molecule, and that the interaction of A1AT with IgE is dependent on non-covalent interactions that are disrupted by anionic detergents during electrophoresis, and as seen with the interaction between A1AT and fibrinogen^48^. Our SEC analysis with a commercial preparation of monomeric A1AT incubated together with recombinant IgE failed to reveal complexes indicative of *in vitro* association between the two proteins (Fig. S5B). This indicates that some other property or constituent of plasma was required for their formation. As clotting removes IgE-tp from serum^9^, fibrinogen may be a candidate, especially as A1AT has recently been shown to be the most abundant non-covalently bound protein associated with fibrinogen^48^.

The forms of A1AT giving rise to dimers most likely bind IgE-tp and IgE-c on the kappa-light chain, which also ends in a cysteine residue (sequence KSFN**RG**D**C**) and is already known to form complexes with A1AT^28^. Lambda-light chains whose C-terminal cysteine residue is penultimate (as in the tailpieces of IgA and IgM) rather than ultimate (as in IgE-tp and kappa light chains) were less able to interact with A1AT^28^. The location of the cysteine residue may therefore determine the nature of the bonding formed with A1AT dimers.

Oligomers of A1AT can arise through multiple independent mechanisms that have been revealed by crystallographic studies, e.g. interaction between the RCL and strand 1C and between the RCL and strand 7A of A1AT (reviewed in^30^,^49^). Such linkages between native monomers are not stable in aqueous solution and are not believed to be the mechanism by which pathological polymers assemble. Alternative linkages have also been proposed including via a β-hairpin of the RCL and strand 5A, and a trimer of A1AT in which strands 1C, 4B and 5B of one molecule are replaced by those of a donor^31^,^32^. Which of these mechanisms of polymerization occur with IgE is unclear, although we believe that the multimeric forms of A1AT that associate with IgE are different in their abundance to those formed by heating A1AT *in vitro* (Fig. 4).

To exclude the possibility of experimental binding artifact, we performed reciprocal immunoprecipitations of A1AT and identified IgE by western blotting with IgE-specific antibodies, whereas an irrelevant isotype-matched control monoclonal antibody (B10) did not pull down either IgE or A1AT (Fig. 2B). The IgE pulled down by either monoclonal antibodies 367 or ATZ11 runs at ~190 kDa, the expected molecular weight for monomeric IgE, and HMW forms of IgE-tp were not pulled down. Given that mAb 367 blocks the formation of HMW forms of A1AT (Fig. S4) it is reasonable to assume that the epitope seen by 367 on the tailpiece is no longer available for binding when IgE-tp is complexed to A1AT, and therefore 367 would only precipitate non-complexed IgE-tp monomers. This also suggests that the HMW forms of IgE-tp seen by direct blotting of plasma with 367 may arise through alternative mechanisms that do not necessarily involve A1AT (Fig. 1). Given the presence of multiple HMW IgE-tp complexes of varying size among individuals (Fig. 1), it is also possible that different complexes of IgE-tp with A1AT can be assembled, and that in some of these structures the tailpiece epitope seen by 367 is still available for binding.

Although dimer and tetramer forms of A1AT clearly bind at different positions on IgE, the mechanism of binding of either form is presently unknown. Low concentrations of guanidine hydrochloride are known to induce A1AT polymer formation^39^,^49^,^50^, and therefore the guanidium groups found within two arginine residues located in the eight amino acid tailpiece (sequence ESS**RR**GGC) may be involved in forming hydrogen bonds with A1AT that also encourage polymer formation. Arginine glycine repeats are already known to facilitate protein-protein interactions that include self-associations^51^.

The high population frequency of the Z allele of A1AT suggests that it confers a selective advantage in affected individuals^35^, and A1AT from a PiZ genotyped individual was still capable of binding IgE (Figs 2B and 3). A major cause of death and chronic morbidity in the pre-drug era was infectious disease, including those caused by helminth parasites, and so the survival benefit may be explained by an exuberant granulocyte influx caused by pro-inflammatory effects of A1AT polymers that protected individuals from parasitism^52^. The PiZ mutation arose 2,000 years ago in the Viking population of southern Scandinavia^53^ that are known to have been heavily parasitized by helminths that reside in the liver^54^.

There is evidence that PiZ heterozygotes without demonstrable lung disease display an airway IL-8 related neutrophilic inflammation^55^. This inflammation would be expected to enhance immune responses and help eradicate tissue dwelling worms. There is evidence from animal models that some immunity to schistosome and hookworm larval migration may occur in the lungs and is highly dependent on both IL-8 and infiltrating granulocytes^22^. However increased longevity and the widespread adoption of cigarette smoking, the main cause of lung inflammation, has the opposite effect. In this case inflammation associated with polymers exacerbate tissue destruction, rather than eradicating parasites. It has been shown that levels of IgE, IL-8 and A1AT rise dramatically after infection with helminth parasites^56^. Indeed A1AT is known to bind directly to schistosomes^57^, is upregulated in the livers of infected animals^58^, and infections with schistosomes aggravate liver disease^59^. Thus A1AT may protect IgE from the damaging effects of serine proteases derived from migrating parasites. It is notable that helminth mediated tropical pulmonary eosinophilia syndrome, in which IgE levels are raised significantly, also leads to acquired A1AT deficiency^60^,^61^.

Cleavage of the reactive centre loop (RCL) of A1AT by a target enzyme, typically elastases, results in a striking conformational transition that ‘flips’ the enzyme from the upper pole of A1AT to the lower pole, essentially trapping the enzyme in a molecular ‘mouse-trap’ for clearance from the site of inflammation^49^,^62^. By pre-treating plasma with varying concentrations of neutrophil elastase, prior to incubation with IgE, we could show that this ‘sprung’ form of A1AT was no longer capable of interacting with IgE, and that therefore the forms of A1AT bound to IgE were still fully functional with respect to enzyme inactivation (Fig. 4). A similar observation has recently been made for the activity of A1AT bound to fibrinogen^48^.

To test this hypothesis, we allowed recombinant IgE-tp to bind A1AT in plasma, prior to incubating the complex with schistosome proteases that we had previously shown cleave IgE-tp at the solvent exposed Cε3/Cε4 inter-domain region of IgE-Fc^26^. We observed that the IgE-tp-A1AT complex was fully protected from proteolytic cleavage by elastase (Fig. 5B). That all the oligomeric forms of A1AT bound to IgE had interacted with elastase was evident by the detection of A1AT reactive bands at 47, 35 and 5 kDa, that are indicative of ‘sprung’ forms of A1AT-protease complexes that remain attached to IgE (Fig. 5C)^42^,^63^. This shows that IgE-tp is protected from the damaging effects of proteases by non-covalently bound A1AT. That IgE and A1AT^56^ are elevated in helminth-infected donors suggests that binding of IgE to A1AT may confer significant advantages to this population (supplemental Fig. S1), although what impact the association of A1AT with IgE has on immunity to helminth parasites now needs to be determined.

**Figure 7:**
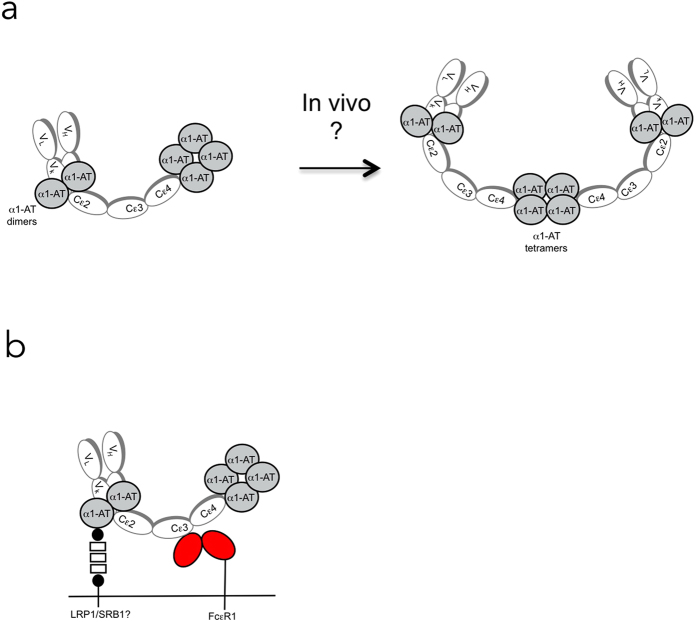
Schematic model for the interaction with A1AT with IgE-tp. (**a**) A1AT can bind at two distinct sites on IgE-tp including the C-terminus of the kappa light chain (dimers) and the C-terminal tailpiece. Binding of A1AT by IgE protects the molecule from proteolytic breakdown by proteases released from parasites or degrading immune cells. The presence of the tailpiece additionally allows for the formation of polymers of A1AT (tetramers) as seen in previous structural studies^30^. We postulate (shown by question mark) that the inherent polymerization ability of A1AT may therefore also drive the polymerization of IgE-tp *in vivo* and that other constituents of plasma may be involved. (**b**) Complexes of A1AT to IgE-c and IgE-tp may allow for cross-linking of FcεR1 with other receptors for A1AT e.g. LRP1 and/or SRB1, that are also expressed on the surface of immune cells, including mast cells, monocytes and dendritic cells.

We had previously shown that cleavage of human IgE by schistosome derived proteases rendered the antibody molecule unable to interact with U937 cells expressing the low-affinity FcεRII^25^. We were therefore interested to determine what effect the presence of A1AT might exert on interactions of IgE with the high affinity FcεRI. We observed that IgE-opsonized beads that had first been incubated in plasma formed significantly greater numbers of rosettes with FcεRI expressing CHK1E1 cells than IgE-beads that had not been incubated in plasma (Fig. 6). Although this effect may not be dependent on A1AT, we believe this to be unlikely, as CHO-K1 cells express receptors for A1AT, including the low density lipoprotein receptor-related protein 1 (LRP1) and scavenger receptor class B (SRB1) that are 98% and 80% identical to human LRP1 and SRB1 respectively (http://www.chogenome.org). Therefore the increased binding seen with IgE-opsonized beads complexed with A1AT may arise from additional cross-linking of IgE-A1AT to LRP1 and/or SRB1 in addition to FcεRI (Fig. 7b). Whatever the role of individual IgE-receptors, our finding that A1AT protects IgE from proteolytic attack without compromising its functional ability would clearly be advantageous during eosinophil-, basophil- or mast cell-mediated degranulation when IgE must resist breakdown by the very proteases it has triggered from these cells. It will also be important to determine the functional consequences of IgE-A1AT-antigen complexes on mast cell activation.

Both LRP1 and SRB1 are expressed on the surface of monocytes and dendritic cells that also express FcεRI^64^,^65^,^66^,^67^,^68^. Human FcεRI on dendritic cells has been shown to contribute to IgE clearance, and cross-linking of IgE/FcεRI on dendritic cells induces immune regulatory responses that diminish allergic responses *in vivo*^5^,^6^. The half-life of infused A1AT is in the region of 4–5 days^69^, which is longer than human IgE (two days), and therefore IgE-A1AT complexes may have even shorter half-life than A1AT alone^70^,^71^.

Greer *et al*. recently described a mechanism for clearance of IgE that involves monovalent ligation of FcεR1 by IgE on dendritic cells and monocytes, that results in internalization and degradation of IgE in endo/lysosomal compartments^8^. It will be important to determine if IgE-A1AT-antigen complexes can be internalized into DCs and monocytes, and what the consequences of internalization are on cellular function, e.g. in affecting antigen presentation.

To explore if there might be a relationship between A1AT and FcεR1 we interrogated the UCSC Gene Sorter (http://genome.ucsc.edu/goldenpath/help/hgNearHelp.html) and Immunet (http://immunet.princeton.edu) databases that help identify functional relationships between genes and proteins. A search of both databases with SERPINA1 revealed such a potential association with human FcεR1. As polymers of A1AT are believed to be pro-inflammatory^52^,^55^, their binding to IgE may allow for faster clearance from sites of inflammation by FcεR1 expressed by dendritic cells and monocytes.

FcεRI is also expressed by platelets and is involved in platelet-mediated cytotoxicity reactions against *S. mansoni*^72^. FcεRI induces platelet aggregation through platelet endothelium aggregation receptor 1 (PEAR1), an interaction that is inhibited by IgE^73^. Anti-IgE therapy with omalizumab has raised concerns of an increased risk of arterial thrombotic events, particularly myocardial infarction (MI) and stroke^74^, conditions that have also been associated with A1AT deficiency (reviewed in^75^). What role IgE-A1AT complexes therefore play in the interaction between FcεRI and PEAR1, and the consequences for platelet mediated cytotoxicity against helminth parasites now need to be determined.

Thus the observation that IgE can be complexed with A1AT may have important consequences for anti-IgE and A1AT supplementation therapies used in the treatment of allergic asthma and COPD respectively. In summary, the novel identification of A1AT binding to IgE-tp uncovers a potential new function for A1AT in the regulation of IgE-mediated immunity and may have important implications for the pathophysiology of pulmonary disease in A1AT-deficiency and other conditions with an increased protease burden.

## Methods

### Antibodies

Human chimeric anti-dansyl IgE-c and IgE-tp stable transfectants were kindly provided by Professor Sherie Morrison, UCLA (University of California, Los Angeles). Cell lines were maintained and IgE antibodies purified from culture supernatants as previously described^9^,^15^,^26^. Monoclonal antibody 367, specific for the C-terminal eight amino acids of IgE-tp, was a kind gift from Professor Andrew Saxon and Dr Ke Zhang (University of California, Los Angeles). Monoclonal anti-human IgE Cε2-specific (clone name 4.15) was kindly provided by Professor Hannah Gould (King’s College, London). Anti-human IgE Cε3 specific monoclonal 8E/4F4 (Biodesign). Anti-mouse IgG conjugated to alkaline phosphatase (Pierce) or peroxidase (Dako) was used to detect monoclonal antibodies B10, 367, 4.15, 8E/4F4 and ATZ11 (Pierce). Monoclonal ATZ11 recognizing a neo-epitope in polymerized A1AT has been described previously^34^ and was kindly provided by Professor Noor Kalsheker (University of Nottingham). Sheep anti-human A1AT-peroxidase was obtained from Serotec. Monoclonal antibody B10 has been previously described^76^. A World Health Organization IgE standard (15,000 IU/ml) was obtained from the Scripps laboratories.

### Plasma

Fresh heparinized blood (20 ml) was collected from healthy human volunteers by venepunture in vacutainers containing lithium-heparin (BD, Biosciences). Blood was transferred to Universal tubes containing a 5 ml solution of 6% dextran T70 (Amersham, Little Chalfont, UK) in 0.9% saline and red cells allowed to sediment for 30 mins at 37 °C. The resultant buffy coat was centrifuged at 1500 g for 5 min to remove leucocytes and remaining plasma stored at −80 ^o^C before using. Plasma samples from Piida, Uganda have been characterized in a previous study^77^. Informed consent was obtained in accordance with the Ugandan Ministry of Health whose ethical review committees approved all protocols. Plasma from hyper-IgE typed individuals was kindly provided by Professor Michael Kerr (University of Leeds). Plasma from a PiZ genotyped individual or from donors with COPD was kindly provided by Professor Noor Kalsheker (University of Nottingham).

### Preparation of dansyl-BSA conjugates

Briefly, 1.2 g of bovine serum albumin (BSA) was dissolved in 35 ml of 0.2M NaHCO_3_, pH8.5. 0.25 g of dansyl chloride (Sigma) was dissolved in di-methyl formamide (DMF), and both solutions were mixed together at room temperature under gentle rotation for 2 h before dialysis against five liters of phosphate buffered saline (PBS) pH 7.4.

### Preparation of dansyl-BSA-sepharose and purification of recombinant IgE

Cyanogen bromide activated sepharose (Amersham) was swollen with 1 mM HCl. 10 mg/ml of dansyl-BSA was diluted in coupling buffer (0.1 M NaHCO_3_, 0.5 M NaCl, pH 8.3) and gently agitated overnight together with the sepharose, and then washed with excess coupling buffer to remove unconjugated hapten, followed by blocking of reactive groups with 0.1 M Tris-HCl buffer, pH 8.0 for 2 h. The sepharose was then washed with alternating high (0.1 M Tris-HCl, 0.5 M NaCl pH 8.0) and low (0.1 M acetate buffer, 0.5 M NaCl, pH 4.0) buffers to remove non-covalently bound material before storage in 20% ethanol. Recombinant IgEs were affinity purified from large volumes (~3 litres) of tissue culture supernatant using dansyl-BSA-sepharose packed columns using an AKTA FPLC frac-950 system (Amersham). Bound IgE was eluted off the column with 0.2 M sodium phosphate pH2 into 100μl of 0.7 M sodium phosphate pH11 to neutralize and eluted IgE containing fractions pooled, concentrated and dialyzed against PBS. Concentrations of purified IgE were determined by bicinchoninic acid (BCA) protein assay kit (Pierce), and purity determined by SDS-PAGE on 4–15% gradient gels.

### Immunoprecipitation

For precipitation studies, typically 50μl of human plasma was incubated with 50μg of antibody for two hours prior to precipitation with 50μl dansyl-BSA-sepharose slurry (in the case of recombinant IgEs) or sheep anti-mouse IgG coated Dynabeads M-280 (in the case of monoclonal antibodies) in a final volume of 0.5 ml of PBS for 1 h. Beads were pelleted by gentle centrifugation and washed five times in 1.5 ml PBS. Up to 80μl of non-reducing or reducing sample buffer was then added to the pelleted beads and heated at 100 ^o^C for 5 min prior to loading at 20μl per lane on 4–15% gradient gels. Proteins were transferred to nitrocellulose membranes (Schleicher & Schuell) and blocked with PBS/0.05% Tween-20 (PBST) containing 5% non-fat milk powder for 1 h under gentle agitation at room temperature before washing 3 times each for 5 minutes with PBST. Blots were cut and incubated in PBST containing a 1:500 dilution of peroxidase-conjugated anti-human A1AT (Serotec or Dako) or alkaline phosphatase-conjugated anti-human IgE (Sigma) for 1 h in PBST. Monoclonal antibodies 367, 4.15, 8E/4F4 or ATZ11 were incubated with blots at 1:500 in PBST prior to washing and detection with peroxidase-conjugated or alkaline phosphatase-conjugated anti-mouse IgG (Pierce). After washing blots were developed in DAB reagent or BCIP/NBT solution according to manufacturers instructions. For experiments investigating the role of the RCL, plasma was incubated with varying concentrations of human neutrophil elastase (Sigma) prior to precipitation of A1AT with recombinant IgE as described above. Resistance of IgE-tp A1AT complexes to degradation by *S. mansoni* cercarial elastases (CE) was determined by Western blotting with monoclonal 367 as described previously^26^.

### FcεRI binding assay

Fifty microliter of dansyl-sepharose beads were opsonized with up to 500μg/ml of recombinant IgE-c or IgE-tp antibodies making use of their specificity for the hapten dansyl. After three washes in PBS, IgE-opsonized beads were incubated in the presence or absence of varying dilutions of plasma for 1 h prior to five further washes in PBS. IgE-opsonized beads were then incubated with the FcεRI expressing CHK1E1 transfectants grown as described previously to confluence in twenty-four well plates^43^. Total numbers of adhering beads were counted and results expressed as a percentage of binding seen with IgE-c in the absence of incubation with plasma.

### Ethical Permission

Full informed consent was obtained for all procedures, and ethical approval for use of anonymized plasma samples was obtained from the Department of Genetics, University of Nottingham ethics review board or the respective institutions at the time of sampling.

## Additional Information

**How to cite this article**: Quinn, P. M. *et al*. IgE-tailpiece associates with α-1-antitrypsin (A1AT) to protect IgE from proteolysis without compromising its ability to interact with FcεRI. *Sci. Rep*. **6**, 20509; doi: 10.1038/srep20509 (2016).

## Supplementary Material

Supplementary Information
