# Supplementary material for: IgE-tailpiece associates with α-1-antitrypsin (A1AT) to protect IgE from proteolysis without compromising its ability to interact with FcεRI
Source: Sci Rep. 2016 Feb 4;6:20509. doi: 10.1038/srep20509 (PMC4740804; doi:10.1038/srep20509)
Supplement: Supplementary Information [file srep20509-s1.pdf]

# Supplementary information for

# IgE-tailpiece associates with α-1-antitrypsin (A1AT) to protect IgE from proteolysis without compromising its ability to interact with FcεRI

**Phyllis M. Quinn[1], David W. Dunne[2], Shona C. Moore[3], and Richard J. Pleass[3]**

[1]Institute of Genetics, School of Biology, University of Nottingham, Nottingham NG7 2UH, UK.
[2]Department of Pathology, University of Cambridge, Tennis Court Road, Cambridge CB2 1QP, UK.
[3]Department of Parasitology, Liverpool School of Tropical Medicine, Pembroke Place, Liverpool, L3 5QA, UK.

Supplementary Fig. 1

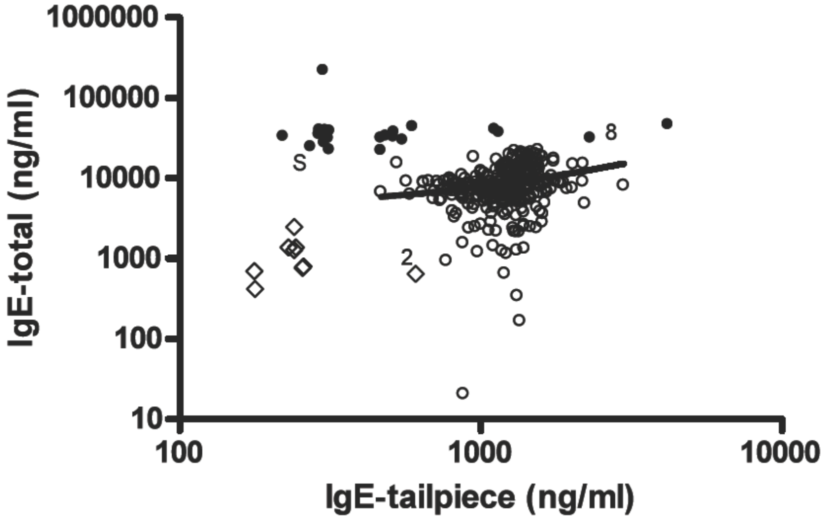


Fig. S1. Plasma concentrations of total IgE-tp or total IgE from healthy UK controls (open diamonds, n = 9), UK hyper-IgE controls (filled circles, n = 21) or Ugandans from an area endemic for schistosomiasis (open circles, n = 380). Each point represents individual plasma IgE concentrations (ng/ml) determined by sandwich ELISAs (described below) with a 12-point standard curve of recombinant IgE-tp or IgE-c as internal standards titrated on the same plate. Median values for each cohort are UK controls (total IgE-tailpiece = 240 ng/ml, vs total IgE = 800 ng/ml), UK hyper IgE (total IgE-tailpiece = 466 ng/ml vs total IgE = 33,783 ng/ml) and Ugandans (total IgE-tailpiece = 1,246 ng/ml vs total IgE = 7,914 ng/ml). Numbers 2 and 8 indicate donors whose plasma contained elevated levels of the tailpiece variant and S indicates the WHO Scripps hyper IgE internal standard used throughout. Numbered plasmas were used in Western blotting experiments presented in Fig. 1. Data represent median values of duplicate wells from two repeated experiments with internal standards run on every plate. For the Ugandan cohort a least squares regression line is shown for reference.

Method: Nunc maxisorp microtiter plates were coated with capturing monoclonal antibodies 367 or 8E/4F4 at 5 µg/ml in carbonate buffer, pH 9.6, and blocked with phosphate buffered saline containing 0.1% Tween-20 (PBST) / 5% non fat milk powder. After 5 washes with PBST, 100µl RPMI/10 % FCS containing appropriately diluted recombinant IgE or human plasma were added and incubated overnight at $4^{o}$C. Following five further washes, the detecting antibody, typically anti-human epsilon heavy chain-HRP (Serotec) diluted 1:1000 in RPMI/10% FCS was added to each well and incubated for two hours. Following washes as above the plate was developed using TMB substrate (Sigma 3, 3', 5, 5'-tetramethylbenzidine tablets made up according to the manufacturer's instructions) and the reaction stopped using 2M $H_2SO_4$ and the plate read at 450nm.

Supplementary Fig. 2

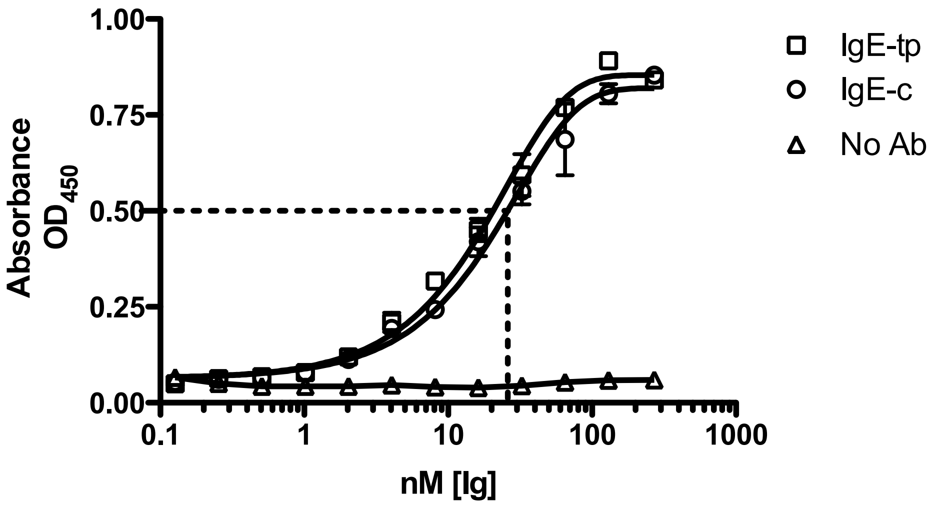


Fig. S2. Recombinant IgE-tp and IgE-c bind human A1AT.

ELISA plates were coated in purified human A1AT (Sigma A9024) at 50μg/ml in carbonate buffer pH9.6 overnight. Plates were blocked in phosphate buffered saline (PBS) containing 0.1% Tween-20 (PBST) / 0.25% bovine gelatin (Sigma) for two hours at room temperature. After three washes in PBS, recombinant IgE-c or IgE-tp were added at the indicated dilutions and incubated for one hour at room temperature. After three washes in PBST/0.25% gelatin, diluted dansyl-BSA-biotin (1:1000 dilution of 1mg/ml stock) in PBS/T was added to each well and allowed to bind for one hour at room temperature. After three further washes a 1:1000 dilution of peroxidase-labeled streptavidin (Serotec) was added and plates incubated for a further hour at room temperature prior to washing as above. Plates were developed using TMB substrate (Sigma 3, 3’, 5, 5’-tetramethylbenzidine tablets made up according to the manufacturer’s instructions) and the reaction stopped using 2M $H_2SO_4$ and the plate read at 450nm.

Supplementary Fig. 3

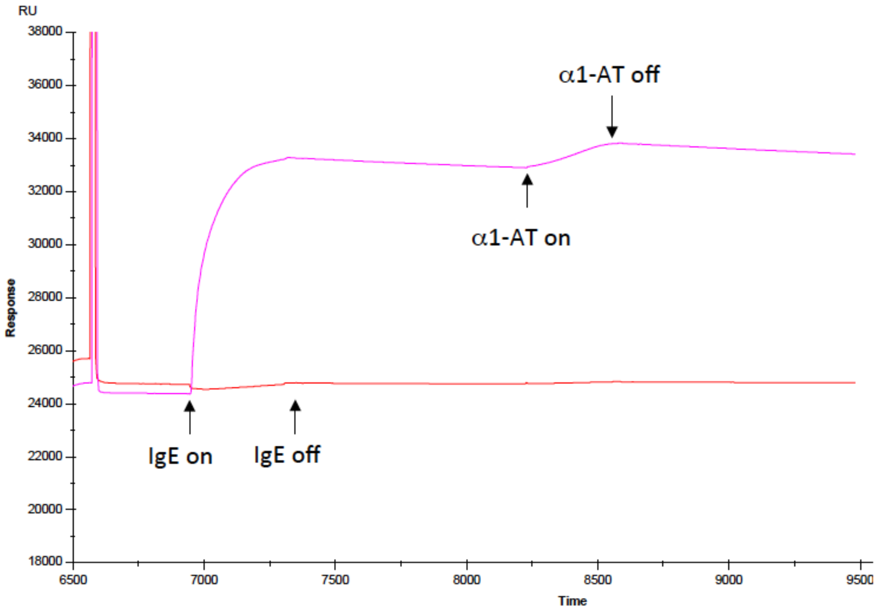


Fig. S3. Surface plasmon resonance (SPR) analysis of A1AT binding to recombinant IgE-tp (pink trace). Dansyl-BSA (2μg) was amine-coupled to flow cells on a CM5 chip according to manufacturers instructions (Biacore AB, Stevenage, UK). IgE-tp was then injected at 10μg/ml in phosphate buffer with a flow rate of 20μl per min and association and dissociation observed over indicated time points in seconds. Purified human A1AT (Sigma) was then injected at 100μg/ml in PBS and association and dissociation from IgE observed. A1AT binding in the absence of IgE is shown by the red trace. We were unable to calculate binding kinetics because of the multiple forms of A1AT that bind at multiple sites on IgE, and are therefore only able to show an interaction between the two molecules by SPR.

Supplementary Fig. 4

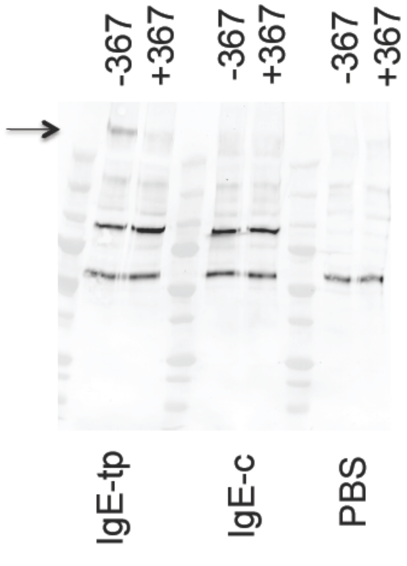


Fig. S4. The tailpiece-specific monoclonal antibody 367 inhibits binding of higher order forms of A1AT to IgE (arrowed) while having no observable effect on the binding of dimeric forms of A1AT.

50μl of dansyl-BSA conjugated to sepharose beads were opsonized with 5μg of IgE-c or IgE-tp for 1h at room temperature. IgE-opsonized beads were then washed in PBS prior to incubation with an excess (50μg) of mAb 367. Beads were washed five times in PBS to remove unbound antibodies and then suspended in 50μl plasma overnight at room temperature. After five washes in PBS, beads were suspended in 100μl of non-reducing sample buffer, heated at 95°C for 5 min, and 20μl run into 4-15% acrylamide SDS-PAGE gradient gels. After transfer to nitrocellulose, blots were incubated with 1 in 500 dilution of peroxidase-conjugated anti-A1AT and developed as described in methods.

Supplementary Fig. 5

A)

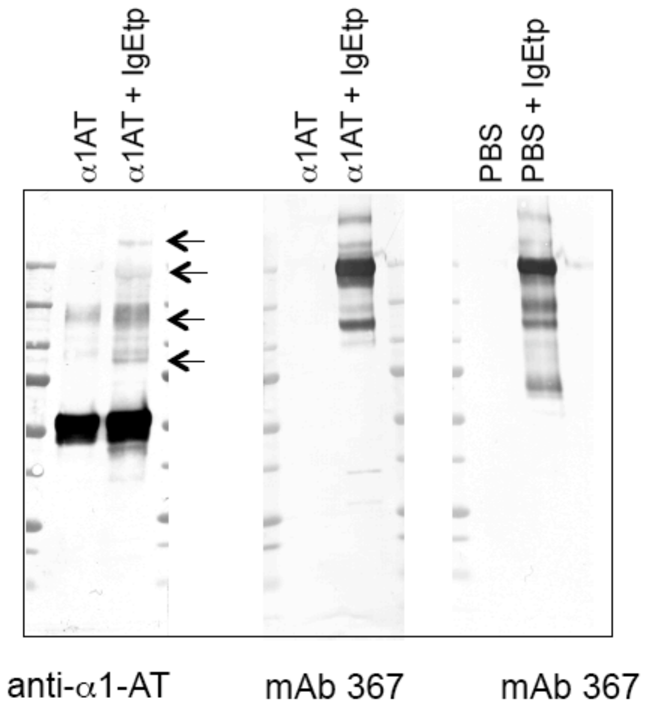


B)

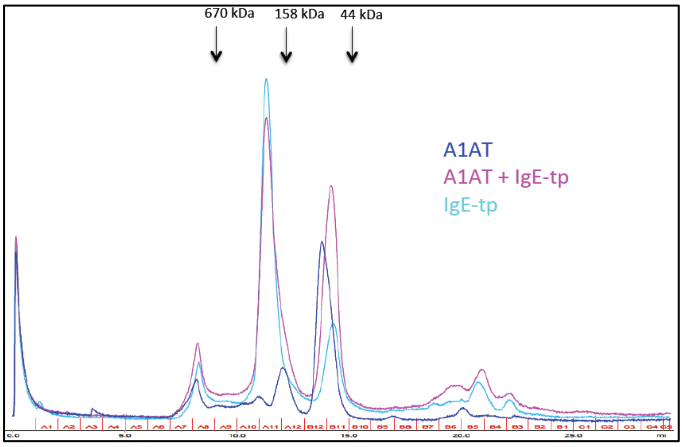


Fig. S5. Monomeric A1AT polymerizes in the presence of IgE-tp. (A) 1.35μg of a size-excluded commercial preparation of monomeric human A1AT (Sigma) or PBS was incubated with 5.5μg of IgE-tp or PBS for 18 hours at room temperature. Samples were then added directly to 10μl of non-reducing sample buffer (no heating) and run directly into SDS-PAGE gradient gels prior to transfer to nitrocellulose membranes and Western blotting with anti-A1AT or anti-tp specific mAb 367 (as described in methods). (B) Samples from A were also subjected to size-exclusion chromatography (SEC) analysis on Superdex-200 10/300GL column showing profiles for A1AT (blue trace), A1AT + IgE-tp (magenta trace), IgE-tp alone (cyan trace). Elution profiles of molecular weight standards are indicated.
